# Identification and Characterization of New Variants in *FOXRED1* Gene Expands the Clinical Spectrum Associated with Mitochondrial Complex I Deficiency

**DOI:** 10.3390/jcm8081262

**Published:** 2019-08-20

**Authors:** Sofia Barbosa-Gouveia, Emiliano González-Vioque, Filipa Borges, Luis Gutiérrez-Solana, Liesbeth Wintjes, Antonia Kappen, Lambert van den Heuvel, Rosaura Leis, Richard Rodenburg, María Luz Couce

**Affiliations:** 1 Diagnosis and Treatment of Congenital Metabolic Diseases Unit (UDyTEMC), Department of Pediatrics, Clinical University Hospital of Santiago de Compostela, 15706 Santiago de Compostela, Spain; 2 Faculty of Medicine, University of Santiago de Compostela, 15706 Santiago de Compostela, Spain; 3 IDIS-Health Research Institute of Santiago de Compostela, 15706 Santiago de Compostela, Spain; 4 Unit of Child Neurology, Department of Pediatrics, Hospital Infantil Universitario Niño Jesús de Madrid, 28009 Madrid, Spain; 5 Department of Paediatrics, Radboud Centre for Mitochondrial Medicine, Radboud University Medical Centre, 6525 GA Nijmegen, The Netherlands; 6 Unit of Pediatric Gastroenterology and Nutrition Department of Pediatrics, Hospital Clínico Universitario de Santiago de Compostela, 15706 Santiago de Compostela, Spain; 7 CIBER Fisiopatología Obesidad y Nutrición (CIBEROBN), Instituto Salud Carlos III, 28029 Madrid, Spain; 8 CIBERER, Pabellón 11, 28029 Madrid, Spain

**Keywords:** mitochondrial disorders, complex I deficiency, FOXRED1, epilepsy

## Abstract

Complex I (nicotinamide adenine dinucleotide (NADH): ubiquinone oxidoreductase) is the largest complex of the mitochondrial oxidative phosphorylation system (OXPHOS) system. Forty-four subunits encoded in nuclear and mitochondrial genomes compose this multiprotein complex, its assembly being a highly complex process involving at least 15 additional nuclear encoded assembly factors. Complex I deficiency is a mitochondrial disorder usually associated with early-onset severe multisystem disorders characterized by highly variable clinical manifestations. Flavin adenine dinucleotide (FAD)-dependent oxidoreductase domain-containing protein 1 (FOXRED1) is a complex I assembly factor. To date, only five patients with mitochondrial complex I deficiency due to mutations in *FOXRED1* have been characterized. Here, we describe a child with ataxia, epilepsy and psychomotor developmental delay carrying two heterozygous *FOXRED1* variants, c.920G>A (p.Gly307Glu) and c.733+1G>A. We demonstrate the molecular mechanism supporting the pathogenicity of the FOXRED1 variants, showing a clear deficiency of complex I activity. The reduction in the steady-state level of complex I holoenzyme in patient fibroblasts, confirmed the pathogenicity of the variants and showed the molecular mechanism behind their pathogenicity. A comparison of the clinical presentation of the index case with the previously described cases allowed deepening our knowledge about the clinical variability associated with *FOXRED1* defects.

## 1. Introduction

Mitochondrial diseases (MDs), caused by defects in the mitochondrial oxidative phosphorylation system (OXPHOS), are one of the most common inborn errors of metabolism with a birth prevalence of 1/5000 [1,2]. Among these, complex I deficiency (MIM #252010) is the most common defect, accounting for 1/3 of all cases [2].

The OXPHOS system, composed of five multi-subunit enzyme complexes and two electron carriers (ubiquinone and cytochrome c), is the main cellular source of energy through ADP phosphorylation to ATP using the electrochemical proton gradient generated by electron transport [3]. Mitochondrial complex I, the major entry point for electrons to the respiratory chain [4], is the first and largest complex of the OXPHOS system [5], and it consists of 44 different subunits codified by nuclear and mitochondrial genes [6,7]. This complex plays a central role in energy metabolism: it has been implicated in the regulation of reactive oxygen species (ROS) and is suggested as the rate-limiting step in overall respiration [4]. Complex I assembly is proposed to involve a stepwise process with intermediate complexes shared by two assembly pathways: de novo synthesis headed by mtDNA-encoded subunits and the dynamic exchange of newly imported nDNA-encoded subunits with pre-existing components of the mature complex [8,9,10].

The complicated assembly of so many subunits requires a number of assembly factors that are not part of the final structure of complex I. Currently, at least 15 known or putative complex I assembly factors have been described and variants in nine of these were associated with isolated complex I deficiency due to impaired complex I biogenesis [9,11,12]. One of those assembly factors is FOXRED1, a 486-amino acid FAD-dependent oxidoreductase. Initially, phylogenetic profiling and subsequent knockdown studies identified FOXRED1 as a candidate protein for complex I biogenesis [13]. Later, Formosa et al. confirmed its role not only in the assembly but also in the stability of late stages complex I intermediates [14].

Complex I deficiency is involved in early-onset severe multisystem disorders [15,16] and represents the biochemical phenotype for ~30% of mitochondrial disease pediatric patients [16]. The clinical symptoms that arise are usually highly heterogeneous, with a poor prognosis, and rapid progression [17]. To date, only five patients with mitochondrial Complex I deficiency due to mutations in *FOXRED1* have been described [18,19,20,21,22]. All the variants identified are located in the same protein domain. Here, we demonstrate the molecular mechanism supporting the pathogenicity of the *FOXRED1* variants found in compound heterozygosity in our patient. Also, we compare the particular clinical presentation of the patient with the previously described cases, expanding the phenotype spectrum associated with *FOXRED1* defects.

## 2. Experimental Section

This study was developed in collaboration with the University Clinical Hospital of Santiago de Compostela (Spain) and Radboud University Medical Center (Netherlands). Parents have provided written informed consent for study participation and the publication of the results. All experimental protocols were approved by the Radboud University Medical Center and were performed in accordance with relevant guidelines and regulations.

### 2.1. Clinical Profile

Our index case, Patient 1, a second child from nonconsanguineous parents, showed intrauterine growth restriction (IUGR) from the first trimester of pregnancy. At 2 months, he was admitted to the hospital for bronchiolitis. From that moment, his parents noticed he was less reactive. Magnetic Resonance Imaging (MRI) of the brain was normal. At 3 months of age, the patient began to have paroxysmal episodes and later showed a language delay. At 2 years old, he showed 5/6 episodes per day of “blanking out” for a short period, with or without clonic movements. He was controlled with oxcarbazepine. In the following year, he presented with loss of muscle tone and started to decline. At 4 years old, he showed loss of awareness and responsiveness episodes with a fixed gaze; had clonic movements of the right leg, 30–40 episodes of sudden falling per day and ataxia. Lactic acidosis was present even without exercise. Currently, at 15 years old, the hypomotor episodes are still present. He has some difficulties adapting to new situations. He is more alert and more responsive but clumsier and more uncoordinated with motor movements (complete clinical history in Supplementary Table S1).

### 2.2. Targeted Next-Generation Sequencing

The patient’s DNA was isolated from lymphocytes and analyzed with targeted next generation sequencing (NGS) panels for mitochondrial diseases. We designed a multi-gene panel consisting of 150 nuclear genes coding for respiratory chain complex subunits and proteins involved in the OXPHOS system function previously reported in the literature.

The genetic data was analyzed through NGS technology consistent in enrichment with an in-solution hybridization technology (Sure Select XT; Agilent Technologies) and subsequent sequencing in the Miseq platform (Illumina). A custom Sure Select probe library was designed to capture the exons and exon–intron boundaries of the targeted genes [23]. Sequence capture, enrichment, and elution were performed according to the manufacturer’s instructions. Image analysis and processing of the fluorescence intensities in sequences (“Base Calling”) was performed with Real-Time Analysis (RTA) software v.1.8.70 (Illumina), and quality control of the data was developed with FastQC v0.10.1 program. Reads were aligned to the reference genome GRCh37 with BWA v0.7.9a [24]. NGSrich v0.7.5 software [25] was used as a control previous to variant detection, and BEDTools 2.17.0 [26] and Picard 1.114 [27] for intermediate steps. VarScan v.2.3.6 [28] and SAMtools v0.1.19 [29] were the variant detection software used for indels and single nucleotide polymorphisms (SNP), respectively and Annovar for variant annotation [30].

To achieve a reliable clinical interpretation of the variants detected, we applied prioritization criteria to predict their pathogenicity according to American College of Medical Genetics and Genomics (AMCG) guidelines [31].

### 2.3. Protein Modeling

The *FOXRED1* gene encodes a 486-amino acid FAD-dependent oxidoreductase domain-containing protein. The protein has a cleavable N-terminal mitochondrial targeting sequence which was shown to be localized to the mitochondrion [32] and associated with the matrix face of the mitochondrial inner membrane [14].

The FOXRED1 (Uniprot AC: Q96CU9; ID: FXRD1_HUMAN) protein FASTA sequence was used to build a protein model generated by SWISS-MODEL. The SWISS-MODEL template library (SMTL version 2019-02-28, PDB release 2019-02-22) was searched with BLAST (Basic Local Alignment Search Tool) [33] and HHBlits (Markov models (HMMs)–based lightning-fast iterative sequence search) [34] for evolutionary-related structures matching the target sequence. By performing this homology model, we tried to determine how the missense mutation detected in both patients may change the protein structure, which most probably leads to local conformational changes inducing an important impact on its function.

### 2.4. Cell Culture

Patient and healthy control fibroblasts were cultured in M199 medium (Gibco) supplemented with 10% *v*/*v* fetal calf serum (FCS) and 1% *v*/*v* penicillin/streptomycin (Gibco) at 37 °C with 5% CO_2_.

### 2.5. Mitochondrial Isolation

Fibroblast pellets from both patients and control cell lines were resuspended in ice-cold 10 mM Tris-HCl, pH 7.6. Cells were then disrupted in a Potter–Elvejhem homogenizer at 1800 rpm, and sucrose was added to make the samples isotonic (250 mM). The homogenized cellular samples were centrifugated for 10 min at 600× *g* and the mitochondria pellets were obtained after centrifugation of the supernatant for 10 min at 14,000× *g*.

### 2.6. Respirometry and OXPHOS Activity

The Seahorse XFe96 Extracellular Flux analyzer (Seahorse Bioscience, Billerica, MA, USA) was used to measure Oxygen Consumption Rate (OCR) and the Extracellular Acidification Rate (ECAR). In the day prior to the assay, control and patient fibroblasts were seeded at 10,000 per well in cell culture medium (M199 supplemented with 10% FCS and 1% pen/strep) and incubated overnight at 37 °C with 5% CO_2_. On the assay day, the cell culture medium was replaced by Agilent Seahorse XP Base Medium with 10 mM glucose (Sigma, St. Louis, MO, USA), 1 mM sodium pyruvate (Gibco), and 200 mM L-glutamine (Life sciences) and then incubated for one hour at 37 °C without CO_2_. Baseline cellular OCR was measured eight times followed by four measurement cycles after the addition of the following inhibitors: 1 µM oligomycin A (Sigma), 2.0 µM and 4.0 µM carbonyl cyanide 4-(trifluoromethoxy) phenylhydrazone FCCP (Sigma), and 0.5 µM rotenone and 0.5 µM antimycin A (Sigma), respectively. After OCR measurements, the cell medium was removed and replaced by 0.33% Triton X-100, 10 mM Tris-HCl (pH 7.6). Seahorse plates were stored at −80 °C and thawed afterward. To measure citrate synthase, 3 mM acetyl-CoA, 1 mM DTNB, and 10% Triton X-100 were added. Citrate synthase activity was measured spectrophotometrically, at 37 °C, using a Tecan Spark spectrophotometer. Measurements were based on the absorption at 412 nm by the product thionitrobenzoic acid (TNB), and citrate synthase activity was calculated from the rate of dithionitrobenzoic acid (DTNB) conversion in the presence of oxaloacetate. OCR was measured before and after the addition of inhibitors and normalized to citrate synthase activity according to Srere et al. [35].

The enzymatic activities of complexes I–V, citrate synthase and protein were assayed spectrophotometrically as previously described [36]. All assays were performed in duplicate using a Konelab 20XT auto-analyzer (Thermo Fisher Scientific, Waltham, MA, USA).

### 2.7. SDS-PAGE and BN-PAGE Immunoblot Analysis

For the detection of FOXRED1 protein and the native mitochondrial complex I in our patient and control cell lines, SDS-PAGE and blue native page immunoblot analysis were performed, respectively. Mitochondrial fractions from patient and control cell lines were used. For SDS-PAGE, samples were heated and denaturated at 70 °C in the presence of β-Mercaptoethanol and separated on a 10% sodium dodecyl sulfate-polyacrylamide gel. For blue-native PAGE, the native mitochondrial complexes were solubilized with 2% *w*/*w* n-dodecyl β-d-maltoside. A total of 15 ug and 25 ug of solubilized mitochondrial protein were separated on a 6–16% precast Native PAGE Bis-Tris gels (Invitrogen). Subsequently, proteins were transferred to Immuno-Blot Polyvinylidene difluoride (PVDF) membranes (0.20 μm Immobilon-P (Millipore IPVH00010)) and immunodetection was performed with the following antibodies: Rabbit polyclonal FOXRED1 antibody (Proteintech:24595-1-AP); CI-NDUFa9 (ab14713, Abcam, Cambridge, UK); CII-SDHA (Ab14715; Abcam, Cambridge, UK); Secondary antibodies goat anti-mouse (P0047; DAKO) and goat anti-rabbit (A00160, Genscript, Piscataway, NJ, USA). The chemiluminescence signal was visualized using the enhanced chemiluminescence kit (ECL, Thermo Fischer Scientific) and the Chemidoc XRS+ system (Biorad, Hercules, CA, USA).

## 3. Results

### 3.1. Molecular Genetics and in Silico Analysis of FOXRED1 Variants

**Figure 1:**
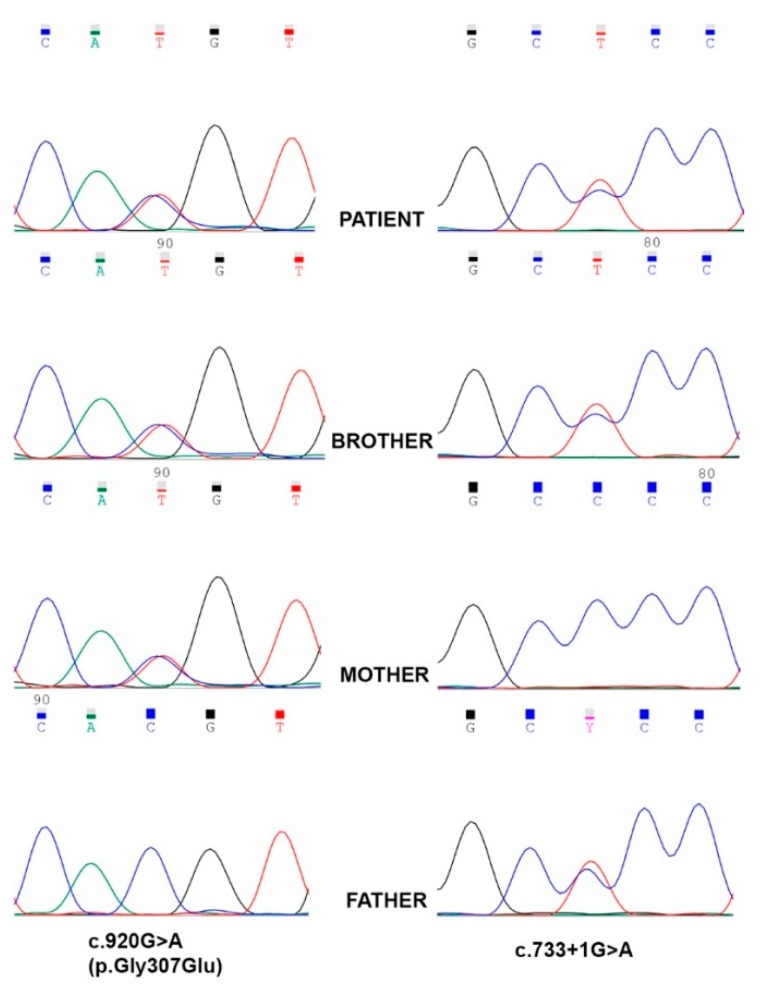
Reverse sequence chromatograms showing Sanger sequencing results. The missense variant c.920G>A (p.Gly307Glu), located in exon 8, was inherited from the mother and the splicing variant, c.733+1G>A, located in intron 6, was inherited from the father. The patient and affected brother harbored both variants in FOXRED1 in a compound heterozygous condition.

Through NGS analysis, two compound heterozygous variants in FOXRED1 (NM_017547.3) were identified in our patient (Figure 1). The variant c.920G>A (p.Gly307Glu), previously reported associated with mitochondrial complex I deficiency, and a splicing variant, c.733+1G>A, not reported so far in human genetic variation databases. Segregation studies were performed through Sanger sequencing to determine the inheritance pattern, and the results are shown in Figure 1. The missense variant was inherited from the mother, and the splicing variant was inherited from the father. The patient has an older brother who also inherited both FOXRED1 variants and shows a similar, although milder, clinical phenotype (clinical history for both patient and brother, patients 1 and 2 respectively, in Supplementary Table S1).

The FOXRED1 variants were analyzed in silico to determine the evolutionary conservation, predicted pathogenicity, functional consequences and Minor Allele Frequency (MAF) within the population (Supplementary Table S2). According to Genomic Evolutionary Rate Profiling (GERP), PhyloP, and phastCons, both variants are in positions of the FOXRED1 protein which are highly conserved through evolution. Pathogenicity was predicted as disease causing by MutationTaster and damaging by FATHMM (Functional Analysis through Hidden Markov Models) and DANN (Deleterious Annotation of genetic variants using Neural Networks) scores. The missense variant c.920G>A is located in exon 8 and results in an amino acid change from a non-polar glycine to an acidic negatively charged glutamic acid at residue 307 and it was predicted as tolerated by SIFT (Sorting Intolerant From Tolerant) and damaging by Provean. The splicing variant c.733+1G>A, located in intron 6, was predicted to destroy the wild-type splice donor site, thus most probably affecting splicing.

### 3.2. FOXRED1 Protein Modeling

**Figure 2:**
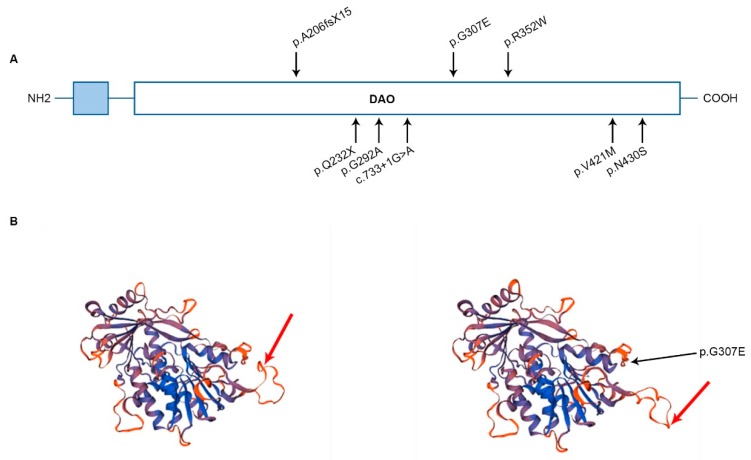
(**A**) Domain structure of FOXRED1 with all the variants identified in the described cases. In light blue, the cleavable mitochondrial targeting sequence is shown or displayed. DAO—FAD-dependent oxidoreductase. (**B**) FOXRED1 protein modeling wild-type and FOXRED1 protein modeling with the mutation p.G307E, reflecting the predicted consequences in the spatial protein structure due to the change of the non-polar amino acid Glycine to the negatively charged Glutamic acid.

All the variants identified so far are located in different regions of the protein and are displayed in the FOXRED1 domain structure (Figure 2A). In Figure 2B, model structures for FOXRED1 with and without the mutation c.920G>A (p.Gly307Glu) are represented. As shown in the image, highlighted with a red arrow, it seems that the missense mutation which induces the change of the non-polar amino acid Glycine to the negatively charged Glutamic acid, displaces the spatial protein structure. In this specific region of the protein loop (red arrow), positively charged amino acids such as Arginine and Lysine are present, and non-polar methionine. The quaternary structure of FOXRED1 changes induced by p.G307E mutation can impact the protein function and, therefore, the susceptibility for disease.

### 3.3. Mitochondrial Respiration and OXPHOS Activity

**Figure 3:**
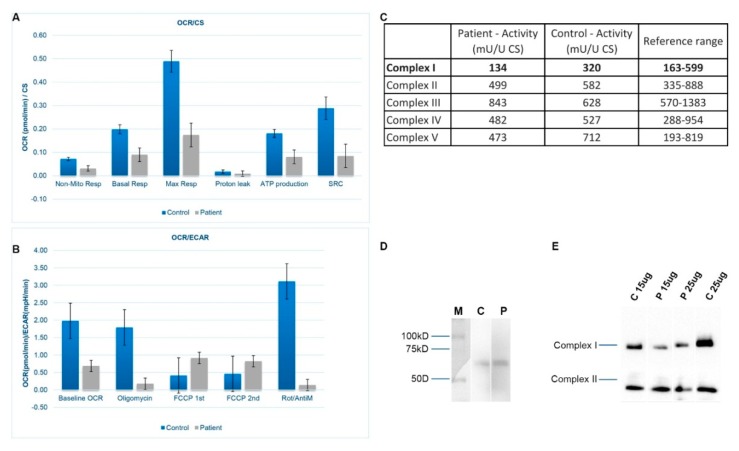
(**A**) Oxygen Consumption Rate (OCR) is measured before and after the addition of inhibitors. The Seahorse XF Cell Mito Stress Test uses compounds of respiration that target components of the Electron Transport Chain (ETC) in the mitochondria to reveal key parameters of metabolic function. These modulators are ETC inhibitors (oligomycin, FCCP, and a mix of rotenone and antimycin A) which were serially injected to measure ATP (adenosine triphosphate) production, maximal respiration (Max Resp), non-mitochondrial respiration (Non-Mito Resp), proton leak, spare respiratory capacity (SRC) and basal respiration (Basal Resp). (**B**) The basal energy metabolism of each cell line was assessed by analyzing OCR/ECAR ratios through sequential injections of the inhibitors. (**C**) Measurement of enzyme activities for the different oxidative phosphorylation system (OXPHOS) complexes in patient and control fibroblasts. FOXRED1 migrated at its predicted size of 54 kDa. CS—Citrate synthase. (**D**) SDS-PAGE immunodetections of FOXRED1 in control and patient fibroblasts. (M) Marker, (C) Control, and (P) Patient. Blot was cropped from different parts of the same gel to show results from samples of interest. A full-length gel is included in Supplementary Material. (**E**) One-dimensional Blue-Native polyacrylamide gel electrophoresis (BN-PAGE) analysis of complex I in gel activity and western blot immunodetection showing differences in complex I amount between control (C) and patient (P) fibroblasts. Blot was cropped from different parts of the same gel to show results from samples of interest. A full-length gel is included in Supplementary Material.

Measurements of OCR and ECAR were performed as well. Significant reductions of both OCR and ECAR in our patient cell line were observed, indicating reduced electron flow through the respiratory chain (Figure 3A,B). These results reflect the deficiency of the cumulative proficiency of the whole set of mitochondrial respiratory chain complexes in patient fibroblasts. Spectrophotometric analysis of respiratory chain enzyme activities revealed an isolated deficiency of complex I (82% of the lowest value of the control range) in fibroblasts from the patient with normal activities of complexes II, III, IV, and V (Figure 3C).

### 3.4. SDS-PAGE and BN-PAGE Immunoblot Analysis

In Figure 3D, the presence of FOXRED1 protein is shown by SDS-PAGE. Immunoblot analysis of one-dimensional Blue-Native polyacrylamide gel electrophoresis (BN-PAGE) gels showed a marked reduced steady-state level of complex I holoenzyme in patient fibroblasts mitochondria (Figure 3E). Different amounts of samples were used, 15 and 25 ug, respectively, and in both cases, there is a clear deficiency of complex I in fibroblasts cells when comparing to control cell lines.

## 4. Discussion

In this work, we have presented the data supporting the pathogenicity of the compound heterozygous *FOXRED1* variants identified in our patient and his brother, in which the splicing mutation c.733+1G>A is reported for the first time. The molecular characterization using the patient’s fibroblasts clearly demonstrated a mitochondrial dysfunction due to abnormal mitochondrial respiration with a significant decrease in the OCR/ECAR ratio, OXPHOS activity and reduced complex I. The genetic results of all the cases described so far, including those presented in this work, are consistent with a recessive mode of inheritance. All the *FOXRED1* pathogenic variants identified lead to a loss of complex I activity and mitochondrial disease.

Patients who show complex I deficiency due to defects in nuclear genes usually have a severe clinical presentation and are associated with early age of death [11]. Pathogenic variants in *FOXRED1*, a nuclear gene which encodes an assembly factor of complex I, seem to challenge this observation. Most of the patients harboring *FOXRED1* pathogenic variants survived to late childhood and adulthood (Supplementary Table S1). Formosa et al. suggested that variants in this gene can result in FOXRED1 partial loss of function and the pathogenic variants are probably hypomorphic [14]. However, Apatean et al. have recently described a clinical case of a female patient, with two variants in the *FOXRED1* gene (c.612_615dupACTG (p.Ala206fsX15) and c.874G>A (p.Gly292Arg)) who died at 3 months with severe pulmonary hypertension and lactic acidosis [21]. From the five patients reported in the literature, Calvo et al. and Apatean et al. give a detailed description of the patients’ clinical presentation, and, as is expected in complex I deficiency, both presented congenital lactic acidosis [19,21]. Our index case and his brother have shown not only a later onset of clinical symptoms but also a later onset of lactic acidosis and the latter has shown mild lactic acidosis as a result of exercise. They both show a less serious clinical presentation in comparison with the previously published cases. Their psychomotor development was not as severely affected as the reported patients. In addition, besides sharing the same *FOXRED1* pathogenic variants, both siblings show great clinical differences between them regarding their quality of life. The youngest, our index case, has lower muscular tone, latent strabismus of the right eye, fewer repetitive crises, low schooling, needs to nap for hours and takes 9 g of bicarbonate per day plus supplements. The older brother has a normal life, with no muscular or visual difficulties, with a normal school attendance although with certain behavioral alterations and learning disorders and he never had a crisis. This clinical variability on disease severity associated with *FOXRED1* mutations can be interpreted as a result of incomplete penetrance, a residual function of the FOXRED1 protein and/or other factors controlling gene expression.

Furthermore, FOXRED1 has been proposed to be a dual function protein: an assembly factor for complex I biogenesis and a key enzyme of amino acid metabolism [37]. As a member of the D-amino acid oxidases, FOXRED1 is functional as an oxidoreductase, and it is hypothesized that this protein can participate in glycine metabolism, which in turn modulates glutathione biosynthesis, an antioxidant protecting the cells from Reactive Oxygen Species (ROS) [32,38]. As previously stated, complex I is the major entry point for electrons into the respiratory chain and also a major producer of ROS species. Mitochondrial disorders are often conditions of oxidative stress (e.g., Leigh syndrome, epilepsy) and symptoms can arise as a result of a functional deficiency that has an assembly factor, reducing complex I activity with a significant increase in ROS species, and, on the other hand, as a result of metabolic activity deficiency, leading to glutathione depletion and preventing the correct cellular ROS detoxification. It could be that depending on the mutation location in *FOXRED1*, the impact on the quaternary structure of the protein and, therefore, in its loss of function, can affect complex I assembly and/or metabolic activity of the ROS species in different forms, influencing the clinical severity spectrum of these disorders. Further studies are needed to elucidate how both functions could be interacting with the molecular mechanism of the disorders associated with FOXRED1 dysfunction.

Overall, in this work we describe two cases of complex I deficiency due to *FOXRED1* pathogenic variants and discuss how *FOXRED1* variants may cause a broad clinical spectrum and be associated with different levels of disease severity—from the severe clinical presentation of patient 6, a three-month-old girl who died from disease complications to the mild presentation of patient 2, the brother of our index case. We demonstrated that the compound heterozygous variants identified were clearly associated with complex I deficiency. More research work is needed to achieve a deep understanding of the relationship between the molecular mechanism and disease progression. This will help in the development of future therapeutic treatments by focusing on specific therapeutic agents, since the responsiveness to a certain treatment may depend on specific gene mutations.

## Supplementary Materials

The following are available online at https://www.mdpi.com/2077-0383/8/8/1262/s1, Table S1: Clinical features observed in the 6 patients described so far harboring pathogenic variants in *FOXRED1*, Table S2: *In silico* analysis to predict FOXRED1 variants pathogenicity according to AMCG guidelines.
