# Supplementary material for: Identification and Characterization of New Variants in FOXRED1 Gene Expands the Clinical Spectrum Associated with Mitochondrial Complex I Deficiency
Source: J Clin Med. 2019 Aug 20;8(8):1262. doi: 10.3390/jcm8081262 (PMC6723710; doi:10.3390/jcm8081262)
Supplement: Supplementary file 1 [file jcm-08-01262-s001.pdf]

Table S1. Clinical features observed in the 6 patients described so far harboring pathogenic variants in *FOXRED1.*

Patient	Variants FOXRED1	Prenatal period	Onset age	Onset clinical symptoms	Evolutionary symptoms									Survival
					Lactic/ Metabolic acidosis	Epilepsy	Muscular tone	MRI	Visual	Respiratory	Cardiovascular	Others	Psychomotor development	
1	c.920G>A (p.Gly307Glu) / c.733+1G>A	IUGR	2m	Hypotonia Development al delay	Yes (+++)	Yes refractory	↓	Normal (2m,4y,7y3m)	Latent strabismus of right eye	Bronchospasm episodes in infant	Normal	AEP normal	IQ: 48	Alive (15y)
2	c.920G>A (p.Gly307Glu) / c.733+1G>A	NI	4y	Clumsiness	With exercise (+)	No	Normal	Normal	Normal	Normal	Normal	Learning disorders	IQ: 99	Alive (19y)
3	c.694C>T (p.Gln232X) / c.1289A>G (p.Asn430Ser)[18]	-	Neonatal period	Premature; Hypoglycemia Congenital lactic acidosis	No (only ↑ lactate in LCR)	Yes	↓	Decreased attenuation in the putamen and cerebellar atrophy (6y)	Normal	Normal	Normal	Normal	Gradually loss of motor skills; wheelchair; no expressive language; understands simple commands	Alive (22y))
4	c.1054 C>T (p.Arg352Trp) / c.1054 C>T (p.Arg352Trp) [19]	NI	Neonatal period	Truncal hypotonia Poor feeding	Yes	Yes	↓	Delayed myelination ventricular dilatation; abnormal signal in the thalami and basal ganglia (8m)	Eye movements have always been roving bilateral optic atrophy	Normal	Mild non-obstructive left ventricular hypertrophy	Persistent hepatomegaly	Psychomotor retardation	Alive (10y)
5	c.1308G>A (p.Val421Met) / c.1308G>A (p.Val421Met)[20]	ND	ND	ND	ND	Yes	NA	NA	NA	NA	NA	NA	Severe psychomotor retardation	Alive (¿)
6	c.612_615dupA CTG (p.Ala206fsX15) / c.874G>A (p.Gly292Arg) [21]	IUGR; Cerebral intraventric ular cysts	Neonatal period	Congenital lactic acidosis. Seizures. Pulmonary hypertension	Yes	Yes	↓	Large periventricular cysts, edematous white matter, delayed myelination (13d)	--	Persistent severe pulmonary hypertension	Dilated right ventricle and atrium; patent ductus arteriosus	-	-	Death (3 months)

IUGR: Intrauterine Growth Restriction; ADHD: Attention Deficit Hyperactivity Disorder; NI: No Incidences; ND: Not Described; MRI: Magnetic Resonance Imaging; IQ: intellectual coefficient; AEP: Auditory Evoked Potentials; y: year; m: month; d: days

Table S2. ***In silico*** **analysis to predict** ***FOXRED1*** **variants pathogenicity according to AMCG guidelines**

Variants FOXRED1 (NM_017547.3)	Inheritance	Population frequencies			Conservation Scores			Prediction Scores			Functional		Splicing	
		**gnomAD**	**ExAC**	**1000 Genomes**	**GERP**	**PhyloP20way**	**phastCons20way**	**DANN**	**Mutation Taster**	**FATHMM**	**SIFT**	**Provean**	**dbscSNV**	**HSF**
c.920G>A (p.Gly307Glu)	Father	0.000152	0.0001	-	5.63	0.953	0.8119	0.9958	DC	D	T	D	NA	NA
c.733+1G>A	Mother	$4.06e^{-5}$	0.00005	-	5.76	0.943	0.9919	0.9926	DC	D	NA	NA	0.999	Break WT donor site

DC: Disease causing; D: Damaging; T: Tolerated; NA: Not Applicable
